# Bioinformatic characterizations and prediction of K^+ ^and Na^+ ^ion channels effector toxins

**DOI:** 10.1186/1471-2210-9-4

**Published:** 2009-03-10

**Authors:** Rima Soli, Belhassen Kaabi, Mourad Barhoumi, Mohamed El-Ayeb, Najet Srairi-Abid

**Affiliations:** 1 Laboratory of Epidemiology and Ecology of Parasites, Institut Pasteur de Tunis, Tunis, Tunisia; 2 Laboratory of Venom and Toxins, Institut Pasteur de Tunis, Tunis, Tunisia; 3 Research and Teaching Building, Institut Pasteur de Tunis, 13 Place Pasteur, BP 74, 1002 Belvedere-Tunis, Tunisia

## Abstract

**Background:**

K^+ ^and Na^+ ^channel toxins constitute a large set of polypeptides, which interact with their ion channel targets. These polypeptides are classified in two different structural groups. Recently a new structural group called *birtoxin*-like appeared to contain both types of toxins has been described. We hypothesized that peptides of this group may contain two conserved structural motifs in K^+ ^and/or Na^+ ^channels scorpion toxins, allowing these *birtoxin-like *peptides to be active on K^+ ^and/or Na^+ ^channels.

**Results:**

Four multilevel motifs, overrepresented and specific to each group of K^+ ^and/or Na^+ ^ion channel toxins have been identified, using GIBBS and MEME and based on a training dataset of 79 sequences judged as representative of K^+ ^and Na^+ ^toxins.

Unexpectedly *birtoxin*-like peptides appeared to present a new structural motif distinct from those present in K^+ ^and Na^+ ^channels Toxins. This result, supported by previous experimental data, suggests that *birtoxin*-like peptides may exert their activity on different sites than those targeted by classic K^+ ^or Na^+ ^toxins.

Searching, the *nr *database with these newly identified motifs using MAST, retrieved several sequences (116 with e-value < 1) from various scorpion species (test dataset). The filtering process left 30 new and highly likely ion channel effectors.

Phylogenetic analysis was used to classify the newly found sequences. Alternatively, classification tree analysis, using CART algorithm adjusted with the training dataset, using the motifs and their 2D structure as explanatory variables, provided a model for prediction of the activity of the new sequences.

**Conclusion:**

The phylogenetic results were in perfect agreement with those obtained by the CART algorithm.

Our results may be used as criteria for a new classification of scorpion toxins based on functional motifs.

## Background

The most-studied components of scorpion venom are polypeptides that recognize ion channels and receptors in excitable membranes, which are harmful to a variety of organisms including human. Two families of toxins that interact specifically with K^+^, and Na^+ ^ion channels, respectively [1] are the subject of intensive work in drug design and development [2-4].

These toxins have been classified according to species-specificity (mammals, insects, and crustaceans), receptor targets (K^+ ^and Na^+^), their lengths (short or long chain), disulfide bonds arrangements [5,6], mechanism of action, and binding site: α or β-like toxins [7,8].

Toxins that affect (modulate) Na^+ ^channels which account for 1 to 10% of raw venom, are long polypeptides with 60–76 amino acid residues [7], reticulated, and stabilized by four disulphide bonds (S-S) [9-11]. Three S-S are located in the molecular core and are conserved across the family while the fourth one is exposed on the molecular surface and varies in position. Considering this characteristic, this disulfide bridge has been named wrapper disulfide bridge [12].

K^+ ^channels toxins are short-chain peptides (22–41 amino acid residues) which are reticulated and stabilized by three or four S-S [13], represent a minor component of the raw venom with the order from 0.05 to 0.1% [14]. In addition, these toxins have particular affinities and specificities for various K^+ ^channel subfamilies [15].

Despite the great variation in the primary structures of many short and long toxins, they share a common structural three-dimensional (3D) conformation [7,14,16,17].

The current available online databases contain up to 800 records of native and mutant toxin sequences enriched with binding affinity, toxicity information, and about 650 3D structures. Scorpion2 [18] and Tox-Prot [19] are two examples of comprehensive database available on the Web.

Recently, a new structural group of toxins with 53–59 amino acids and only 3 S-S, called *birtoxin*-*like *peptides have been characterized [5,6,12,20-24]. This structural group contains peptides with similar sequences that show differences in activity. Some peptides are active on Na^+ ^channels [20,23], while others are active or putatively active on both (K^+ ^and Na^+^) channels [5,6,22].

Given the above characteristics of this new group of toxins, it is clear that classification of K^+ ^or Na^+ ^ion channels effector toxins, based on their lengths and the number of S-S is not fully adequate, to identify correctly the activity of a given toxin. The objectives of this study are:1) identification of signatures (motifs) associated with a given activity on the K^+ ^and/or Na^+ ^channels; 2) verifying the presence of these motifs in the *birtoxin-like *family.

Within this framework, we planned to perform the following steps: sampling of toxins active on K^+ ^and/or Na^+ ^ion channels and determination of the structural signature corresponding to each type of the channels (K^+ ^and Na^+^) effectors. A statistical model (classification model) that uses these motifs and their secondary (2D) structure to predict the function of a given toxin was built.

## Methods

### Sequences preparation and highly similar sequences elimination

Key words "K^+^, Na^+^, channel, scorpion toxin" were used to search the NCBI database [25] which is linked to *swissprot*, *pdb *and *embl *among other databases, for existing K^+ ^and Na^+ ^channel effectors (toxin sequences). Whole length and fragment sequences were included to insure maximum coverage of these toxins with sequence information.

All sequences were gathered according to their activities. Sequences that belong to the same group were put in the same file in FASTA format. Three groups were obtained: the first contains toxins active on the Na^+ ^channels; the second contains toxins active on the K^+ ^channels, and the third contains *birtoxin*-like peptides. A perl script was used to conduct batch PSI-BLAST [26] against the *nr *(non-redundant protein) database to search for similarity and to characterize these toxins.

### Multiple sequence alignment

Multiple sequences alignment was performed using ClustalW [27] version 2 [28]. Multiple alignments of sequences for each group of toxins and for all sequences pooled together, were carried out in order to characterize potential conserved and variable areas for each sequence partition as well as consensus sequences (centroides). To date, ClustalW is still the most popular alignment tool. However, recent methods, in some cases, offer significantly better alignment quality. Thus, this same stage was carried out by another multiple alignments software: CHAOS & DIALIGN [29], which is based on a combination of local and global alignment methodologies. The obtained results were compared with the former ones.

In order to keep random noise at its lowest level, all qualified sequences contained three or four S-S pattern. Based on these results, highly similar sequences were removed to reduce potential bias on the motifs search (motifs conserved in each group). A subset of the originally found sequences, judged representative of the K^+ ^and/or Na^+ ^ion channel effectors, were used as training dataset. This subset reflects a trade-off between sequences that cover most of scorpion species, and sequences that are *centroide *in the multiple alignments. However, for the *birtoxin-like *group, all sequences were sampled.

### Motifs search

Using the training dataset composed of the selected toxins representative of the ion channels blockers for K^+ ^and Na^+^, GIBBS [30,31] version 2.05 and MEME [32,33] version 3.5.4 which is part of the Meta-MEME [34] package, were used to dig out conserved motif information, for all considered sequence partitions.

We used GIBBS to discover the conserved motif(s) (with pre-fixed length, which can contain(s) gaps) specific to each group of ion channel effectors. This same stage was performed by MEME, which determines un-gapped multilevel motifs (with no pre-fixed length). The most significant results were compared and retained.

### Database search for new K^+ ^and Na^+ ^channel effector toxins

The output of these programs (GIBBS and MEME) was thoroughly investigated, and then used as input in the next step of the analysis.

A profile using these motifs was systematically formed, and used to search for sequences with this profile. For that purpose, MAST, which is also part of the Meta-MEME package, was used to search for new K^+ ^and Na^+ ^channel effector sequences (test dataset) against *nr *database. The e-value for a qualified sequence was set to 1. A perl script was used to eliminate replications (identical to the sequences of the training dataset).

### Interproscan

InterPro [35] is an integrated resource for protein families, domains and functional sites, which also integrates a number of protein signature databases as well as the appropriate search tools for them. The motifs found were then searched for in the InterPro database using InterProScan.

### 2D-structure determination

The distribution of the 2D structure in the motif region for each sequence was studied in order to propose a relation linking 2D structure to the function for each group of toxins. The 2D structure of all the sequences (training and test datasets) was determined based on the program PHD [36-38] using neural network approach, and the Softberry's software PSSfinder [39], which uses Markov chains probabilistic model.

### Phylogenetic analysis

Phylogenetic analysis was carried out to:1) study the potential evolutionary relations between the new sequences and already known toxins, 2) infer potential common functions, and 3) classify the new ones (with unknown functions) with respect to the other toxins with known activities.

Since multiple-alignment is at the base of any phylogeny, alignments obtained from ClustalW results were used as entry for program PHYLIP [40] version 3.6 used to build phylogenetic trees. Distances were generated using Jones-Taylor-Thornton model. Phylogenetic trees were constructed using the neighbor-joining algorithm [41].

A construction of the phylogenetic trees for each of sequence group considered was also performed. Consensus trees were determined using the bootstrap method [42].

### Statistical analysis

Correspondence analysis was performed, and biplot [43] was drawn, to illustrate the association between the motifs found and the activities of the toxins from the training dataset. To test the strength of this association, a χ^2 ^test of independence of factors was used where *p-value *was computed using Monte-Carlo simulation [44]. This procedure is implemented in the R software version 2.7 [45], and used in case where large sample theory is not valid (many cell having values less than 5). The same test χ^2 ^was used to measure association between the distribution of the 2D structure (determined by PHD and PSSfinder) of the motifs, and the toxins' activities. In order to arrange the toxins according to their activities (dependent variable) and the motifs determined and their 2D structures (structural variables), classification and regression trees (CART) [46] were built using the procedures implemented in the software Splus version 6.2 [47]. The built classification trees (for each 2D program results) were then used to predict the classes of the new sequences (test dataset), in a purely statistical way independent of the phylogeny results.

## Results

Using the key words "K^+^, Na^+ ^channel inhibitor toxin", about 700 sequences were found from the NCBI server. Among them 495 are confirmed experimentally. No initial filtering was performed to avoid losing any potential signals. All the 495 sequences were used to conduct batch PSI-BLAST one search iteration against the *nr *database. The E-value threshold required for sequence inclusion was set to 10^-5^. After elimination of identical sequences, and those that do not have the S-S pattern, all information on each individual sequence of these potential channels toxins were retrieved. Non-fragment sequences were dug out from the *nr *database, and placed in an Excel file. To prepare for the conserved region search by ClustalW, all sequence partitions were placed in FASTA format in separate files. Based on preliminary ClustalW results, highly similar sequences were removed to reduce bias on conserved, and functionally overrepresented motifs search.

After careful examination of the resulting data, 79 sequences judged as representatives of the class of toxins active on K^+ ^and/or Na^+ ^channels, and confirmed experimentally, were sampled. These 79 toxins (training dataset) covered 18 scorpion species and distributed as follow: 27 toxins active on K^+ ^channels, 38 active on the Na^+ ^channels and 14 toxins belonging to the *birtoxin*-like group. ClustalW re-applied on this training dataset, revealed the following:

1) Eight cysteine residues implicated in the formation of S-S in long toxins were conserved in all Na^+ ^channel effectors except for: CsI, CsvI, and CsvII (probably because of their loop between the second and third β strands, which is longer than the one for other Na^+ ^channel scorpion toxins [48]). 2) Three cysteine residues were conserved in all K^+ ^channel effectors. 3) Five cysteine residues were conserved in the *birtoxin-*like group toxins. 4) One motif: ADVPGNYPL was conserved in this group. 5) For all sequences pooled together, only 3 cysteine residues were conserved, and there is no conserved region (substring).

However, Chaos/Dialign did not reveal any conserved region for all sequence partitions.

### Motifs search results

The training dataset, in its different partitions, was used as input to GIBBS and MEME to conduct motif search. While GIBBS provides gapped or un-gapped motifs, MEME identifies only un-gapped motifs.

### GIBBS results

Because of the variable and the relatively small length of the considered sequences, we opted for a motif length of 10 and 20. Analysis of GIBBS output revealed that there was one motif conserved in all Na^+ ^channel effectors except for CsV, which is considered as structurally intermediate homologous to α and β toxins [49]. A motif conserved for all K^+ ^channel effectors except for two sequences (TsKapa, and TsTxK-α). For the new group of *birtoxin*-like peptides, one motif was conserved. For all sequences pooled together, GIBBS did not identify any particular motif of interest (present in all type of sequences). Similar results were obtained with motif of length 20. However, the motifs obtained using length 10 and 20 respectively were not nested, and they did not overlap (Table 1).

**Table 1:** GIBBS Results.

**Motif Length**	**Group**	**Most Probable Motif**
10	K^+^	AKCMNGKC-CY

10	Na^+^	ACYC--LPE-V-IW

10	*birtoxin-like*	ADVPGNYPLD

20	K^+^	VPCT-SPQCI-PCK-A-M--GKCMNR

20	Na^+^	Q-LGRWGNACYC--LPD-VPIR--G-C

20	*birtoxin-like*	VPGNYPLDKDGNTY-LELGEN

### MEME results

**Figure 1:**
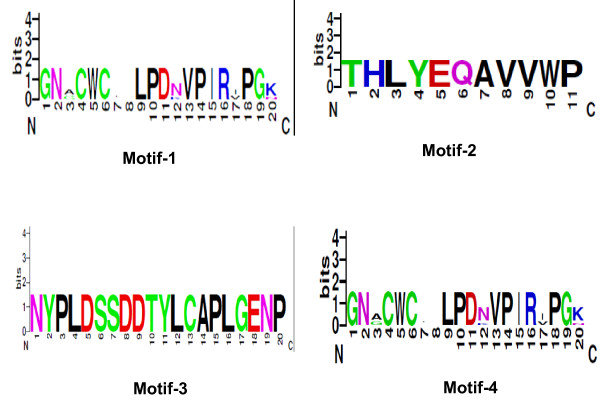
**MEME motifs in logo format**.

Using MEME and considering the relative diversity among K^+ ^and Na^+ ^channel effectors and the length range of these toxins, the maximum motif number was set to 6 (varied from 1 to 6) and maximum motif length was set to 20. Four motif runs were reasonable and provided the best and the most significant distribution of motifs among the groups of toxins. Therefore, we used this run as input to the MAST and Meta-MEME programs. These multilevel motifs are in PSSM format and the consensus (most probable) strings are shown in the Table 2 and Figure 1.

**Table 2:** MEME Results.

**Motif Number**	**Group**	**Length**	**Consensus (Most Probable) Motif**
1	Na^+^	20	GNACWCIELPDNVPIRIPGK
2	Na^+^	11	THLYEQAVVWP
3	*birtoxin-like*	20	NYPLDSSDDTYLCAPLGENP
4	K^+^	20	KDAGMRFGKCMNRKCHCTPK

Motif-1 was conserved in 32 sequences, all of them are α-type Na^+ ^channel blockers. Motif-2 was conserved in 5 sequences, all of them are β-type Na^+ ^channel blockers. Motif-3 was observed in 15 sequences among them 14 toxins belong to the *birtoxin*-like group, and only one is Na^+ ^channel effector: CsvII. Motif-4 was observed in 16 sequences where 14 of them are active on the K^+ ^channels and 2 are *birtoxin*-like peptides (*birtoxin *and *ikitoxin*). The remaining 14 sequences, (1 Na^+ ^channel effector, and 13 K^+ ^channel effectors) did not report statistically significant matches to any of the consensus motifs described in table 2. However, these results do not exclude that these sequences may contain other probabilistic variants of the motifs found. Indeed, 11 among the 13 K^+ ^channel effectors were reported by GIBBS to have a conserved motif of length 20, that overlap with the one identified by MEME (motif4). The comparison of the results obtained by MEME with those obtained by GIBBS, showed that the motifs of length 20 determined by GIBBS were either included in the motifs determined by MEME or significantly overlapped with them. However, the results obtained by GIBBS are less significant in probabilistic terms and in the ability of differentiating among the groups of toxins. Therefore, only motifs obtained by MEME will be adopted for the rest of this work.

### Identification of new K^+ ^and Na^+ ^channel effectors

Using motifs obtained by MEME, we conducted a MAST search against the *nr *database and we analyzed the results. To avoid missing any signal, we have set the maximum motif number to 6, which may lead to an increase in the number of false positives due to model over fitting. Therefore, as a remedy, the e-value for a qualified sequence was set to 1, which may minimize type-I error (false positives). One hundred and seventeen sequences of various scorpion species were retrieved. Not only all the identical sequences but also the ones used as input (training dataset) from the search results were removed. Thus, 72 sequences remained (test dataset). Then, known K^+ ^and Na^+ ^channel effector sequences in the returned test dataset were removed (42 sequences removed). In total, 30 strong candidates as potential new K^+ ^or/and Na^+ ^channel effector toxins were found among five scorpion species. Details are listed in Table 3.

**Table 3:** Result of the activity prediction of the newly identified sequence using phylogenetic analysis, and probabilities of classification by CART tree models adjusted with the motifs and their 2D structure.

**Access Number**	**Inferred Activity by Phylogeny**	**Classification Probability by CART: PSSfinder (PHD)**		
		**Na^+^**	**K^+^**	***birtoxin*-like**

gb|AAF31297.1	**Na^+^**	1	0	0

gb|AAA69557.1	**Na^+^**	1	0	0

gb|AAT36746.1	**Na^+^**	1	0	0

prf||0804800B	**Na^+^**	1	0	0

gb|AAD47376.1	**K^+^**	0	1	0

gb|AAG39641.1	**Na^+^**	1	0	0

prf||0804800A	**Na^+^**	1	0	0

pdb|1LQI|	**Na^+^**	1	0	0

gb|AAF34872.1	**Na^+^**	1	0	0

gb|AAK06898.1	**Na^+^**	1	0	0

gb|AAF31477.1	**Na^+^**	1	0	0

gb|AAA69558.1	**Na^+^**	1	0	0

gb|AAT36745.1	**Na^+^**	1	0	0

gb|AAG00580.1	**Na^+^**	1	0	0

gb|AAP33620.1	**Na^+^**	1	0	0

gb|AAP34332.1	**Na^+^**	1	0	0

gb|AAG09657.1	**Na^+^**	1	0	0

gb|AAG39643.1	**Na^+^**	1	0	0

gb|AAV64254.1	**Na^+^**	1	0	0

gb|AAT52203.1	**Na^+^**	1	0	0

emb|CAD60540.1	**Na^+^**	1	0	0

gb|AAF29465.1	**Na^+^**	1	0	0

gb|AAR08045.1	**Na^+^**	1	0	0

pdb|1SEG|A	**Na^+^**	1	0	0

gb|AAR08044.1	**Na^+^**	1	0	0

gb|AAB31528.1	**Na^+^**	1	0	0

emb|CAD60541.1	**Na^+^**	1	0	0

gb|AAB21461.1	**Na^+^**	1	0	0

gb|AAG39640.1	**Na^+^**	1	0	0

gb|AAB21462.1	**Na^+^**	1	0	0

PSSfinder based tree and (PHD based tree when different).

### Pattern and domain analysis

Pattern and domain analysis were further used to filter the results. All these 30 new K^+ ^and Na^+ ^channel effector sequences have at least one of the 4 conserved motifs previously found in this study. We aligned these 30 sequences and other known K^+ ^and Na^+ ^channel effectors. These motifs were overrepresented among all these sequences (newly identified and known toxins), thus, indicating that the identified motifs in the study are useful for searching potential new K^+ ^and Na^+ ^channel effectors from scorpions or plants.

### InterPro results

Searching InterPro for the identified motifs showed that motif-1 corresponded to 2 hits: PD000908 characteristic of long chain scorpion toxins, and PF00537 corresponding to Toxin_3 domain characteristic of Na^+ ^channel inhibitors from scorpion or plants. Motif-2 and motif-3 did not correspond to any match. Motif-4 corresponded to 2 hits: PD003586 characteristic of short chain scorpion toxins, and PF00451 corresponding to Toxin_2 domain. The fact that 2 motifs among the 4 identified overlap with other already reported in InterPro is a corroboration of our approach.

### 2D results

Using PHD and PSSfinder, all 2D structures of the sequences were determined. PHD results showed that the 2D structures displayed by motif-1, motif-2, motif-3, and motif-4 were mainly β-sheet, α-helix, β-sheet, and α-helix, respectively. Using PSSfinder, the 2D structure motif-1, motif-3, and motif-4 were mainly β-sheet, β-sheet, α-helix, or β-sheet, respectively. The 2D structure displayed by motif-2 was unidentified.

### Phylogenetic analysis results

**Figure 2:**
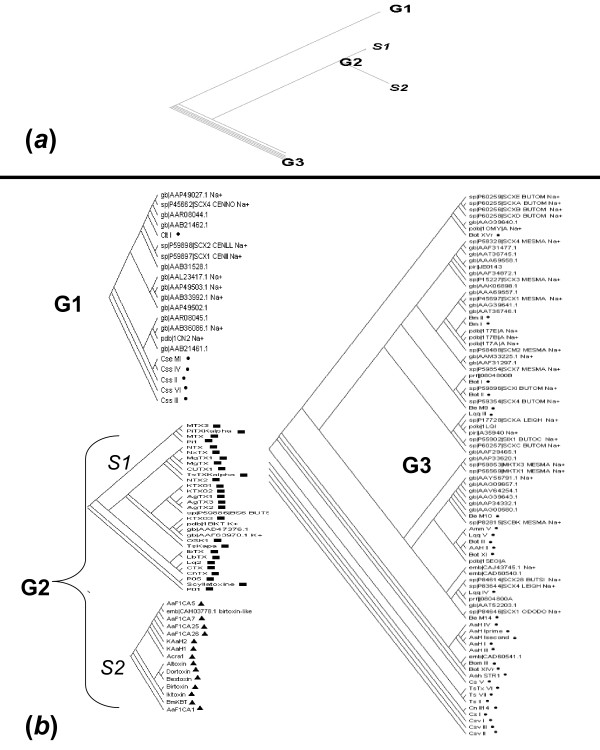
**Consensus phylogenetic tree built using all sequences (training dataset and test dataset)**. *(a) *A Simplified phylogenetic tree that displays three major clades labeled G1, G2, and G3. G2 is further subdivided into two groups S1 and S2. *(b) *Individual clades; G1, S1, S2, and G3. Symbols: – Training dataset sequences: black circle active on Na^+ ^channel, black square active on K^+ ^channel, black triangle *birtoxin-like*. – Test dataset sequences with known activity: (_Na^+^): active on Na^+ ^channel, (_K^+^): active on K^+ ^channel, birtoxin-like: _birtoxin-like.

All 152 sequences (training and/or test datasets) including known and newly identified K^+ ^and/or Na^+ ^channel toxins, found in our analysis, were used to build phylogenetic consensus trees. Using the training dataset only (79 sequences), the consensus phylogenetic tree obtained revealed 3 major groups or clusters (figure 2a):

Group G1; contains all sequences of α-type Na^+ ^channel toxins. Group G2 contains toxins having some activities on K^+ ^channel. This group can be further subdivided into two subgroups: S1 containing toxins active on K^+ ^channel only, and S2 involving *birtoxin*-like peptides. Group G3 contains β-type Na^+ ^channel toxins. The group S2 of *birtoxin*-like peptides was situated between clusters S1 and G3, where S1 contains sequences active on K^+ ^channel and G3 contains β-type Na^+ ^channel toxins. Therefore, S2 can be considered as a transition group from toxins active on K^+ ^channel to those β-type active on Na^+ ^channel (figure 2b). This consensus tree presents the classical divergence between α and β Na^+ ^channel toxins, and it is consistent with previous phylogenetic constructions made by [14,49] even though, they did not use the same set of toxin sequences.

To infer the function of the test dataset toxins (72 sequences), all sequences from training and test datasets were used to build a consensus phylogenetic tree, see figure 2. We examined the whereabouts of the sequences from the test dataset with respect to the clusters (groups) defined by the training dataset. Thus, to perform a final check on the reliability of the results obtained, we examined the whereabouts of the 42 sequences of the test dataset with known function in the output of PHYLIP with respect to the clusters defined by the training dataset (79 sequences). The results show that these sequences (42) with known activities fall within compatible functionally defined clusters (clusters defined by toxins of the training dataset), thus, supporting our results.

The activities of the remaining 31 sequences were inferred in the same manner, see table 3 and figure 1. We can see that most newly identified peptides were grouped with sequences with known function toxins.

### Statistical analysis results

**Figure 3:**
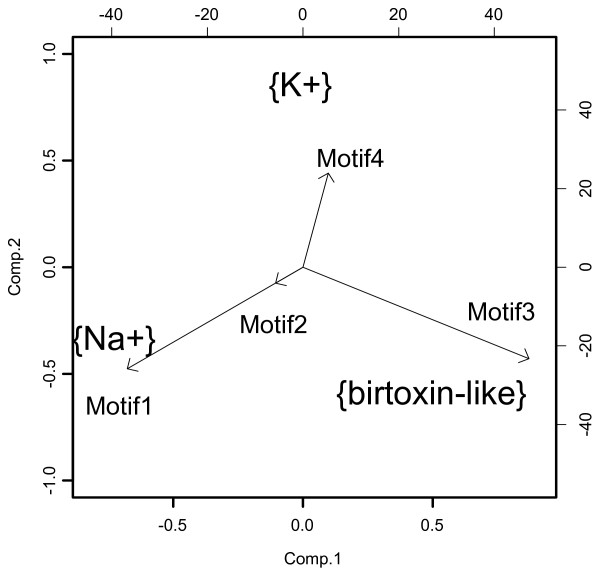
**Biplot, of the correspondence analysis, distribution of the motifs and the toxin functions**.

The association between generated motifs and the toxins activities was very significant, simulated p-value < 0.0001. See biplot figure 3

Similarly, and while the majority of the motifs displayed a β-sheet structure, the association between motifs 2D structure distribution, and the protein classes was evaluated with the same χ^2 ^test, and simulated p-value < 0.001. Therefore, 2D structures of the motifs were incorporated as explanatory variables in the CART model.

Classification trees were adjusted using the training dataset of 79 sequences. Misclassification error rates for PHD and PSSfinder 2D based trees were less of 0.05, indicating good classifications. The same models were used to predict the activities of the 72 sequences identified by MAST. The 42 sequences with known activities were correctly classified, while the remaining 30 new sequences of the test dataset were assigned activities using CART model. The predicted activities were in perfect concordance with the results of the phylogeny. This fact confirms the classification tree models built using the training dataset as well as the results of the phylogeny.

## Discussion and conclusion

Due to the relative stability of K^+ ^and Na^+ ^channel effectors provided by 3 or 4 S-S, they are used as tools for biological investigation of the ion channel structure [50,51], and represent potential candidates for use in medical and pharmacological applications.

Toxins from certain structural family generally target the same receiver with varied intensity. However, in general, each K^+ ^and Na^+ ^channel effector is slightly different from each other, which makes it possible to find suitable toxins for a specific application [52]. This underlines the regular need to identify new K^+ ^and Na^+ ^channel effector toxins.

The analysis of the protein structures based on the conserved motifs is largely used, and it is proven useful in the prediction of the protein functions [53-55].

While no single motif was conserved in all K^+ ^and Na^+ ^channel effector sequences, this approach has permitted the identification of 4 motifs overrepresented and specific to each sequence function/class. Therefore, these motifs may be used as a criteria for the classification of these toxin types, in addition to the usual classification based on sequence length and number of S-S.

Due to the complex nature of the voltage gated Na^+ ^channel, there are many regions of the protein that can be attacked, and therefore it is not surprising that we found two overrepresented motifs in Na^+ ^channel toxins. Interestingly, both motifs (motif-1 and motif-2 for α and β toxins respectively) for Na^+ ^channel toxins found in this study overlap with sites (Hydrophobic face, C-terminal region and β2–β3 strands) described as essential for Na^+ ^channels binding [56-60]. Motif-4 contains amino-acids that are important in K^+ ^toxin activity [61-63].

It was expected that the *birtoxin*-like group (new group) would include both or at least one of the motifs characteristic of K^+ ^and Na^+ ^channel effectors (motif-1, motif-2, or motif-4). However, this was not the case, and toxins of this new group, did exhibit another overrepresented motif (motif-3). This finding suggests that toxins of the *birtoxin-like *group do not interact with the same functional sites as other long or short chains toxins. This hypothesis is supported by competitive binding experiments showing that although KAaH1 (a member of the *birtoxin*-like group) is active on Kv1 channels, it did not displace iodinated α-DTx, sKTX, and CTx (Kv1 channels blockers) from rat brain synaptosomes (Abid, unpublished data). Similarly, AaBTX-L1 which is active on Na^+ ^channel (and also a member of the *birtoxin-like *group), did not compete with ^125^I-CssIV (Na^+ ^channel blocker). Moreover, no competition was observed either with ^125^I-sKTX (K^+ ^blocker) or with ^125^I-Apamine (SK_Ca _channels blocker) on their receptor sites on rat brain synaptosomes [23]. Moreover, *birtoxin*, *ikitoxin*, *dortoxin *and *alitoxin *do not enhance the binding of ["H"]BTX to rat brain synaptosomes, which is not consistent with their action on voltage-gated Na^+ ^current [20].

*birtoxin *and *ikitoxin *were found to contain 2 motifs: motif-3 specific to *birtoxin*-like peptides group and motif-4 which is overrepresented in K^+ ^channel toxins. This suggests that *birtoxin *and *ikitoxin *could interact with K^+ ^channel through this motif. However, this hypothesis needs to be verified by testing these toxins on K^+ ^channels.

The result obtained by the CART algorithm based on the identified motifs and their 2D structures provided an identical classification to that obtained by the phylogeny. Therefore, the knowledge about the motifs and their 2D are sufficient to infer the activity of a given toxin. This conclusion is supported by the fact that binding sites of toxins to their channels target are generally situated on the α-helix or β-sheet 2D structures [22,60,61].

The search for conserved motifs and the phylogenetic analyses enabled us to find common characteristics to each protein family and thus to predict the structure and the function of the new protein sequences. These motifs allowed us to find sequences that we were not able to find with the classical criteria of toxin length and S-S number.

All 30 identified potential K^+ ^and Na^+ ^channel effector toxins possess the overrepresented motifs specific to each group of K^+ ^and/or Na^+ ^channel effector and the S-S domain. While the e-value set for MAST was equal to one, which is a quite stringent criterion, it led to the discovery of very significant motifs with potential biological function (modulating specific ion channels), and provided high sensitivity, and minimized the false positives, with respect to MAST use.

Moreover, the phylogeny has shown that all the newly identified potential K^+ ^and Na^+ ^channel effectors were closely grouped to other known toxins. The new sequences were situated inside the groups limited by toxins with known functions.

In addition, the *birtoxin*-like peptides (S2) were located between cluster containing toxins active on K^+ ^channels (S1) and cluster containing Na^+ ^channel β-type toxins. Therefore, S2 sequences can be considered as a transition group putatively active on both channels (K^+^, and Na^+^). Indeed, this fact explains the presence of both types (K^+^, and Na^+^) of toxins in this group. The challenge will be to find in the *birtoxin*-like (growing in size) group, which are the signatures responsible for K^+ ^or Na^+ ^channel modulation. Unfortunately, this group does not contain till now sufficient sequences, and was not fully characterized with respect to K^+ ^and Na^+ ^channels activities to make a consistent conclusion.

Future work will deal with a finalization of the identified motifs as to discern the exact number of residues, location, and implication for the toxin activities. Docking Study and building biophysical models that incorporate these motifs, and model the interaction with their targets, will be of great use.

## Competing interests

The authors declare that they have no competing interests.

## Authors' contributions

RS performed the sequences extraction produced the training data and performed the analysis. BK performed the statistical analysis and part of the sequence analysis. MB studied the biological significance of the results and performed a critical review of the manuscript. ME co-directed this work and performed a critical review of the manuscript. NSA supervised and co-directed the study, and performed a critical review of the manuscript. All authors contributed significantly in the drafting of the manuscript. All authors have read and approved the manuscript.
